# Supplementary material for: Validity and Reliability of Kinvent Plates for Assessing Single Leg Static and Dynamic Balance in the Field
Source: Sensors (Basel). 2023 Feb 20;23(4):2354. doi: 10.3390/s23042354 (PMC9967360; doi:10.3390/s23042354)
Supplement: Supplementary file 1 [file sensors-23-02354-s001.zip › Parameters_calculation.pdf]

**Parameters calculation : matlab code**

```matlab
%copX : medio-lateral position of the centre of pressure at all times
%copY : antero-posterior position of the centre of pressure at all times


%% Displacement of the CoP
Path_lengthX(i)=abs(copX(i+1)-copX(i));
PLml = mean(Path_LengthX)

Path_lengthY(i)=abs(copY(i+1)-copY(i));
PLap = mean(Path_LengthY)

Path_lengthcop(i)=sqrt(abs((copX(i+1)-copX(i))^2+abs(copY(i+1)-copY(i))^2));
PLcop = mean(Path_Lengthcop)


%% Velocity of the CoP
VelocityX(i)=abs(copX(i+1)-copX(i))/(1/freq);
MVml = mean(VelocityX)

VelocityY(i)=abs(copY(i+1)-copY(i))/(1/freq);
MVap = mean(VelocityY)

Velocitycop(i)=sqrt(abs((copX(i+1)-copX(i))^2+abs(copY(i+1)-copY(i))^2))/(1/freq);
MVcop = mean(Velocitycop)


%% Surface Area
% data = x and y coordinates of CoP
covariance = cov(data);
[eigenvec, eigenval ] = eig(covariance);
SA = pi*prod(2.4477*sqrt(svd(eigenval)));


%% Time to Stabilization

Fz=BW.*9.81;% Vertical Force (N)
One_quarterSD_fz=std(Fz)/4;
Mean_fz=mean(Fz);

Sum_cum_fz=cumsum(Fz);% Cumulative sum of Fz
Sum_cum_size=[];% numbers of element to divided Sum_cum_fz by the Sum_cum_size
Sum_cum_size(1)=1;
Element=1;% Increment

for i=1:length(Fz)-1
   Sum_cum_size(i+1)=Sum_cum_size(i)+Element;
end
```

```
Sum_cum_size=Sum_cum_size';

Cum_Average=Sum_cum_fz./Sum_cum_size;% Cumulative average of Fz (N)

lim_sup_SE = Mean_fz+One_quarterSD_fz;% superior limit
lim_inf_SE = Mean_fz-One_quarterSD_fz;% inferior limit

idx=0;
for i=1:length(Cum_Average)-freq
  count=0;
  if Cum_Average(i)>=lim_inf_SE & Cum_Average(i)<=lim_sup_SE % if the cumulative average find
it between the limits
    for j=i:i+freq-1
      if Cum_Average(j)>=lim_inf_SE & Cum_Average(j)<=lim_sup_SE
        count=count+1;
      end
    end
    if count>=freq %if the cumulative average find it between the limits during 1 second
      idx=i;
      break
    end
  end
end

TTS = abs(Time(idx)-Time(1));
```
