# Supplementary material for: Validity and Reliability of Kinvent Plates for Assessing Single Leg Static and Dynamic Balance in the Field
Source: Sensors (Basel). 2023 Feb 20;23(4):2354. doi: 10.3390/s23042354 (PMC9967360; doi:10.3390/s23042354)
Supplement: Supplementary file 1 [file sensors-23-02354-s001.zip › Table_S1.pdf]

Table S1. Comparison of results obtained with the PLATES vs. AMTI in the laboratory for Single Leg Balance (SLB) and Single Leg Landing (SLL) tests.

			PLATES Lab (mean ± SD)		AMTI Lab (mean ± SD)	ICC (95% CI)	Bias (95% CI)
**SLB**							
PLap	OE	R	258 ± 52,3		244,1 ± 38,3	0,8 (0,49 - 0,93)	13,9 (-118,8 - 146,6)
(mm)		L	241,2 ± 27,5		234,2 ± 43,2	0,74 (0,37 - 0,91)	7 (-117,8 - 131,8)
	CE	R	496,9 ± 70,5	*	556 ± 120,9	0,91 (0,76 - 0,97)	-59,1 (-240,3 - 122,2)
		L	465,7 ± 76,3	*	528,5 ± 111	0,75 (0,39 - 0,91)	-62,8 (-380,8 - 255,2)
PLml	OE	R	256.4 ± 43.6		273.8 ± 40.1	0.79 (0.45 - 0.93)	-17.4 (-130.8 - 96)
(mm)		L	254.7 ± 37.2		256.4 ± 28.8	0.7 (0.28 - 0.89)	-1.7 (-121.7 - 118.4)
	CE	R	493.9 ± 59.5	*	589.1 ± 92.1	0.83 (0.55 - 0.94)	-95.2 (-267.1 - 76.6)
		L	453.1 ± 62.6	*	559.4 ± 80.7	0.75 (0.38 - 0.91)	-106.3 (-310.3 - 97.7)
PLcop	OE	R	403.6 ± 73.5		403.6 ± 55.9	0.82 (0.52 - 0.94)	0 (-173.2 - 173.2)
(mm)		L	388.4 ± 42.8		384 ± 54.6	0.72 (0.34 - 0.9)	4.4 (-180.4 - 189.3)
	CE	R	778.2 ± 91.5	*	897.9 ± 166.5	0.88 (0.67 - 0.96)	-119.7 (-396.1 - 156.7)
		L	722.7 ± 99.7	*	854 ± 137.3	0.76 (0.4 - 0.92)	-131.2 (-526.4 - 263.9)
MVap	OE	R	26 ± 5,3		24,8 ± 3,9	0,79 (0,47 - 0,93)	1,2 (-12,6 - 15)
(mm/s)		L	24,3 ± 2,8		23,8 ± 4,4	0,74 (0,37 - 0,91)	0,5 (-12,2 - 13,2)
	CE	R	50,3 ± 7,2	*	56,5 ± 12,3	0,91 (0,75 - 0,97)	-6,2 (-24,9 - 12,5)
		L	46,8 ± 7,7	*	53,7 ± 11,3	0,76 (0,4 - 0,92)	-6,9 (-38,5 - 24,8)
MVml	OE	R	25.8 ± 4.4		27.8 ± 4.1	0.78 (0.44 - 0.92)	-2 (-13.7 - 9.7)
(mm/s)		L	25.6 ± 3.8		26.1 ± 2.9	0.7 (0.28 - 0.89)	-0.4 (-12.6 - 11.8)
	CE	R	49.8 ± 6.2	*	59.9 ± 9.4	0.83 (0.55 - 0.94)	-10 (-27.6 - 7.5)
		L	45.6 ± 6.3	*	56.9 ± 8.2	0.76 (0.4 - 0.92)	-11.3 (-31.6 - 9.1)
MVcop	OE	R	40.7 ± 7.4		41 ± 5.7	0.81 (0.5 - 0.93)	-0.4 (-18.4 - 17.7)
(mm/s)		L	39.1 ± 4.3		39 ± 5.5	0.72 (0.33 - 0.9)	0.1 (-18.7 - 18.9)
	CE	R	78.4 ± 9.3	*	91.3 ± 16.9	0.88 (0.67 - 0.96)	-12.9 (-40.9 - 15.1)
		L	72.7 ± 10	*	86.8 ± 14	0.76 (0.41 - 0.92)	-14.1 (-53.4 - 25.2)
SA	OE	R	771.9 ± 240.6		582.6 ± 174.9	0.33 (-0.22 - 0.72)	189.2 (-519.7 - 898.1)
(mm^2)		L	680.4 ± 189.1		672.8 ± 293.9	0.73 (0.34 - 0.9)	7.6 (-547 - 562.3)
	CE	R	1861.7 ± 485		2088 ± 914.9	0.81 (0.51 - 0.94)	-226.4 (-1419.8 - 967.1)
		L	1652.9 ± 468.8		2059.9 ± 839.1	0.63 (0.17 - 0.87)	-407 (-2078.6 - 1264.6)
**SLL**							
TTS		R	2.98 ± 0.21	*	3.24 ± 0.36	0.82 (0.53 - 0.94)	-0.26 (-0.72 – 0.20)
(s)		L	2.99 ± 0.17	*	3.31 ± 0.34	0.67 (0.23 - 0.88)	-0.32 (-1.04 – 0.40)
PLcop		R	623.1 ± 87.7		648.6 ± 85.4	0.86 (0.61 - 0.95)	-25.5 (-179.7 - 128.7)
(mm)		L	605.4 ± 53.9	*	667.2 ± 56.6	0.86 (0.63 - 0.95)	-61.8 (-233.5 - 109.8)
MVcop		R	46 ± 6.2		46.5 ± 5.8	0.86 (0.61 - 0.95)	-0.6 (-12.1 - 10.9)
(mm/s)		L	45 ± 4.6		47.3 ± 4	0.88 (0.67 - 0.96)	-2.3 (-14.7 - 10)
SA		R	1016.2 ± 278.5		1098.2 ± 322.9	0.36 (-0.19 - 0.74)	-82 (-707.9 - 543.9)
(mm^2)		L	1002.8 ± 240.7		979.5 ± 235.2	0.5 (-0.02 - 0.8)	23.3 (-622.7 - 669.3)

OE: Open Eyes ; CE: Closed Eyes ; L: Left leg ; R: Right leg; CI: Confidence Interval ; SD: Standard Deviation ; SLB : Single Leg Balance ; SLL : Single Leg Landing ; PLap : anteroposterior Path Length ; PLml : mediolateral Path Length ; PLcop : Centre of Pressure Path Length ; MVap : anteroposterior Mean Velocity ; MVml : mediolateral Mean Velocity ; MVcop : centre of pressure Mean Velocity ; SA : Surface ; TTS : Time To Stabilization ; *: significant difference ($p < 0.05$)
