# Supplementary material for: Validity and Reliability of Kinvent Plates for Assessing Single Leg Static and Dynamic Balance in the Field
Source: Sensors (Basel). 2023 Feb 20;23(4):2354. doi: 10.3390/s23042354 (PMC9967360; doi:10.3390/s23042354)
Supplement: Supplementary file 1 [file sensors-23-02354-s001.zip › Table_S2.pdf]

Table S2. Comparison of results obtained in the laboratory vs. in the field with the PLATES for Single Leg Balance (SLB) and Single Leg Landing (SLL) tests.

			PLATES Lab (mean ± SD)	PLATES Field (mean ± SD)	ICC (95% CI)	Bias (95% CI)
**SLB**						
PLap (mm)	OE	R	258 ± 52,3	262,2 ± 51,8	0,9 (0,71 - 0,97)	-4,2 (-104,6 - 96,3)
		L	241,2 ± 27,5	241,3 ± 40,6	0,88 (0,68 - 0,96)	-0,1 (-75,9 - 75,6)
	CE	R	496,9 ± 70,5	532,9 ± 102,7	0,88 (0,66 - 0,96)	-36 (-259,6 - 187,6)
		L	465,7 ± 76,3	486,6 ± 67,6	0,93 (0,81 - 0,98)	-20,9 (-209,8 - 168)
PLml (mm)	OE	R	256.4 ± 43.6	262.4 ± 33.5	0.84 (0.57 - 0.95)	-6.1 (-97.4 - 85.3)
		L	254.7 ± 37.2	244.1 ± 37.3	0.69 (0.28 - 0.89)	10.6 (-95.1 - 116.4)
	CE	R	493.9 ± 59.5	501.2 ± 68.9	0.82 (0.54 - 0.94)	-7.3 (-176.3 - 161.6)
		L	453.1 ± 62.6	477 ± 59.4	0.81 (0.5 - 0.93)	-23.9 (-199.6 - 151.8)
PLcop (mm)	OE	R	403.6 ± 73.5	412.3 ± 63.2	0.89 (0.7 - 0.96)	-8.8 (-143.8 - 126.3)
		L	388.4 ± 42.8	380.2 ± 56.6	0.80 (0.49 - 0.93)	8.2 (-129 - 145.5)
	CE	R	778.2 ± 91.5	813.1 ± 122.1	0.87 (0.65 - 0.96)	-34.9 (-324.5 - 254.7)
		L	722.7 ± 99.7	757.9 ± 91.8	0.90 (0.73 - 0.97)	-35.1 (-307.5 - 237.2)
MVap (mm/s)	OE	R	26 ± 5,3	26,4 ± 5,2	0,9 (0,72 - 0,97)	-0,4 (-10,4 - 9,5)
		L	24,3 ± 2,8	24,3 ± 4,1	0,89 (0,68 - 0,96)	0 (-7,7 - 7,6)
	CE	R	50,3 ± 7,2	53,9 ± 10,4	0,88 (0,66 - 0,96)	-3,6 (-26,7 - 19,5)
		L	46,8 ± 7,7	49 ± 6,9	0,93 (0,81 - 0,98)	-2,1 (-21 - 16,8)
MVml (mm/s)	OE	R	25.8 ± 4.4	26.5 ± 3.4	0.84 (0.57 - 0.95)	-0.6 (-9.8 - 8.5)
		L	25.6 ± 3.8	24.6 ± 3.7	0.71 (0.3 - 0.9)	1.1 (-9.5 - 11.6)
	CE	R	49.8 ± 6.2	50.6 ± 7	0.81 (0.52 - 0.94)	-0.8 (-18.3 - 16.7)
		L	45.6 ± 6.3	48 ± 6	0.81 (0.5 - 0.93)	-2.4 (-20.1 - 15.2)
MVcop (mm/s)	OE	R	40.7 ± 7.4	41.6 ± 6.3	0.89 (0.7 - 0.96)	-0.9 (-14.3 - 12.5)
		L	39.1 ± 4.3	38.3 ± 5.7	0.81 (0.5 - 0.93)	0.8 (-13 - 14.6)
	CE	R	78.4 ± 9.3	82.2 ± 12.3	0.87 (0.63 - 0.95)	-3.8 (-33.7 - 26.1)
		L	72.7 ± 10	76.3 ± 9.3	0.91 (0.73 - 0.97)	-3.6 (-30.9 - 23.7)
SA (mm^2)	OE	R	771.9 ± 240.6	658.2 ± 240.3	0.87 (0.63 - 0.95)	113.7 (-288.5 - 515.9)
		L	680.4 ± 189.1	660 ± 262.6	0.54 (0.03 - 0.82)	20.5 (-485.7 - 526.6)
	CE	R	1861.7 ± 485	2067.4 ± 634.5	0.77 (0.42 - 0.92)	-205.7 (-1540.4 - 1129)
		L	1652.9 ± 468.8	1891.2 ± 518.9	0.86 (0.62 - 0.95)	-238.3 (-1391.1 - 914.5)
**SLL**						
TTS (s)		R	2.98 ± 0.21	3.03 ± 0.23	0.88 (0.68 - 0.96)	-0.05 (-0.42 - 0.31)
		L	2.99 ± 0.17	2.95 ± 0.17	0.63 (0.18 - 0.87)	0.04 (-0.85 - 0.93)
PLcop (mm)		R	623.1 ± 87.7	624 ± 72.3	0.84 (0.58 - 0.95)	-0.9 (-178.6 - 176.8)
		L	605.4 ± 53.9	627.1 ± 86.1	0.84 (0.57 - 0.95)	-21.8 (-196.5 - 153)
MVcop (mm/s)		R	46 ± 6.2	47.6 ± 5.2	0.83 (0.55 - 0.94)	-1.7 (-15.9 - 12.6)
		L	45 ± 4.6	46.1 ± 6	0.89 (0.69 - 0.96)	-1.1 (-12.9 - 10.7)
SA (mm^2)		R	1016.2 ± 278.5	892.2 ± 281.5	0.07 (-0.46 - 0.56)	124 (-584.7 - 832.7)
		L	1002.8 ± 240.7	957.8 ± 256.3	0.42 (-0.12 - 0.77)	45 (-655.1 - 745.1)

OE: Open Eyes ; CE: Closed Eyes ; L: Left leg ; R: Right leg ; CI: Confidence Interval ; SD: Standard Deviation ; ICC: Intraclass Correlation Coefficient ; SLB : Single Leg Balance ; SLL : Single Leg Landing ; PLap : anteroposterior Path Length ; PLml : mediolateral Path Length ; PLcop : CoP Path Length ; MVap : anteroposterior Mean Velocity ; MVml : mediolateral Mean Velocity ; MVcop : CoP Mean Velocity ; SA : Surface ; TTS : Time To Stabilization.
