# Supplementary material for: Validity and Reliability of Kinvent Plates for Assessing Single Leg Static and Dynamic Balance in the Field
Source: Sensors (Basel). 2023 Feb 20;23(4):2354. doi: 10.3390/s23042354 (PMC9967360; doi:10.3390/s23042354)
Supplement: Supplementary file 1 [file sensors-23-02354-s001.zip › Table_S3.pdf]

Table S3. Comparison of results obtained with PLATES-Field vs. AMTI-Laboratory for Single Leg Balance (SLB) and Single Leg Landing (SLL) tests.

			PLATES Field (mean ± SD)		AMTI Lab (mean ± SD)	ICC (95% CI)	Bias (95% CI)
**SLB**							
PLap	OE	R	262,2 ± 51,8		244,1 ± 38,3	0,72 (0,33 - 0,9)	18,1 (-137,2 - 173,4)
(mm)		L	241,3 ± 40,6		234,2 ± 43,2	0,83 (0,56 - 0,94)	7,1 (-100,2 - 114,4)
	CE	R	532,9 ± 102,7		556 ± 120,9	0,9 (0,72 - 0,97)	-23,1 (-228,8 - 182,7)
		L	486,6 ± 67,6		528,5 ± 111	0,69 (0,27 - 0,89)	-41,9 (-429,1 - 345,3)
PLml	OE	R	262.4 ± 33.5		273.8 ± 40.1	0.78 (0.45 - 0.92)	-11.3 (-123.3 - 100.6)
(mm)		L	244.1 ± 37.3		256.4 ± 28.8	0.63 (0.18 - 0.87)	-12.3 (-131.3 - 106.7)
	CE	R	501.2 ± 68.9	*	589.1 ± 92.1	0.81 (0.52 - 0.94)	-87.9 (-257 - 81.2)
		L	477 ± 59.4	*	559.4 ± 80.7	0.82 (0.54 - 0.94)	-82.4 (-275.6 - 110.8)
PLcop	OE	R	412.3 ± 63.2		403.6 ± 55.9	0.74 (0.37 - 0.91)	8.7 (-194.2 - 211.6)
(mm)		L	380.2 ± 56.6		384 ± 54.6	0.75 (0.39 - 0.91)	-3.8 (-177.4 - 169.8)
	CE	R	813.1 ± 122.1	*	897.9 ± 166.5	0.89 (0.69 - 0.96)	-84.8 (-354.8 - 185.2)
		L	757.9 ± 91.8		854 ± 137.3	0.74 (0.37 - 0.91)	-96.1 (-546.3 - 354)
MVap	OE	R	26,4 ± 5,2		24,8 ± 3,9	0,71 (0,31 - 0,9)	1,6 (-14,3 - 17,6)
(mm/s)		L	24,3 ± 4,1		23,8 ± 4,4	0,82 (0,54 - 0,94)	0,5 (-10,7 - 11,8)
	CE	R	53,9 ± 10,4		56,5 ± 12,3	0,9 (0,71 - 0,97)	-2,6 (-23,9 - 18,7)
		L	49 ± 6,9		53,7 ± 11,3	0,7 (0,29 - 0,89)	-4,7 (-43,2 - 33,8)
MVml	OE	R	26.5 ± 3.4		27.8 ± 4.1	0.78 (0.44 - 0.92)	-1.4 (-12.8 - 10.1)
(mm/s)		L	24.6 ± 3.7		26.1 ± 2.9	0.63 (0.17 - 0.86)	-1.5 (-13.7 - 10.7)
	CE	R	50.6 ± 7	*	59.9 ± 9.4	0.81 (0.51 - 0.94)	-9.2 (-26.4 - 7.9)
		L	48 ± 6	*	56.9 ± 8.2	0.83 (0.56 - 0.94)	-8.8 (-27.9 - 10.2)
MVcop	OE	R	41.6 ± 6.3		41 ± 5.7	0.73 (0.35 - 0.91)	0.6 (-20.3 - 21.4)
(mm/s)		L	38.3 ± 5.7		39 ± 5.5	0.75 (0.37 - 0.91)	-0.7 (-18.8 - 17.3)
	CE	R	82.2 ± 12.3	*	91.3 ± 16.9	0.88 (0.68 - 0.96)	-9.1 (-36.9 - 18.7)
		L	76.3 ± 9.3		86.8 ± 14	0.75 (0.39 - 0.91)	-10.5 (-55.1 - 34.1)
SA	OE	R	658.2 ± 240.3		582.6 ± 174.9	0.26 (-0.3 - 0.68)	75.5 (-667 - 818)
(mm²)		L	660 ± 262.6		672.8 ± 293.9	0.41 (-0.13 - 0.77)	-12.8 (-821.1 - 795.5)
	CE	R	2067.4 ± 634.5		2088 ± 914.9	0.89 (0.7 - 0.96)	-20.6 (-1027 - 985.7)
		L	1891.2 ± 518.9		2059.9 ± 839.1	0.62 (0.16 - 0.86)	-168.7 (-2216.1 - 1878.8)
**SLL**							
TTS		R	3.03 ± 0.23	*	3.24 ± 0.36	0.73 (0.35 - 0.9)	-2.1 (-0.73 – 0.32)
(s)		L	2.95 ± 0.17	*	3.31 ± 0.34	0.75 (0.38 - 0.91)	-0.36 (-1.17 – 0.44)
PLcop		R	624 ± 72.3		648.6 ± 85.4	0.82 (0.53 - 0.94)	-24.6 (-217.3 - 168.2)
(mm)		L	627.1 ± 86.1		667.2 ± 56.6	0.74 (0.37 - 0.91)	-40.1 (-273.7 - 193.5)
MVcop		R	47.6 ± 5.2		46.5 ± 5.8	0.83 (0.56 - 0.94)	1.1 (-13.5 - 15.7)
(mm/s)		L	46.1 ± 6		47.3 ± 4	0.81 (0.51 - 0.94)	-1.2 (-16.4 - 14)
SA		R	892.2 ± 281.5		1098.2 ± 322.9	0.22 (-0.33 - 0.66)	-206 (-889.9 - 477.9)
(mm²)		L	957.8 ± 256.3		979.5 ± 235.2	0.38 (-0.17 - 0.75)	-21.6 (-568 - 524.7)

OE: Open Eyes ; CE: Closed Eyes ; L: Left leg ; R: Right leg ; CI: Confidence Interval ; SD: Standard Deviation ; SLB : Single Leg Balance ; SLL : Single Leg Landing ; PLap : anteroposterior Path Length ; PLml : mediolateral Path Length ; PLcop : Centre of Pressure Path Length ; MVap : anteroposterior Mean Velocity ; MVml : mediolateral Mean Velocity ; MVcop : centre of pressure Mean Velocity ; SA : Surface ; TTS : Time To Stabilization ; *: significant difference ($p < 0.05$)
