# Supplementary material for: Validity and Reliability of Kinvent Plates for Assessing Single Leg Static and Dynamic Balance in the Field
Source: Sensors (Basel). 2023 Feb 20;23(4):2354. doi: 10.3390/s23042354 (PMC9967360; doi:10.3390/s23042354)
Supplement: Supplementary file 1 [file sensors-23-02354-s001.zip › Table_S4.pdf]

Tables S4 : Variation Coefficient obtained with PLATES-Laboratory, PLATES-Field and AMTI for Single Leg Balance (SLB) and Single Leg Landing (SLL)

			Coefficient of Variation (%)		
			PLATES-Lab	*PLATES-Field*	***AMTI-Lab***
**SLB**					
PLap (mm)	OE	R	19,08	17,49	18,17
		L	**12,48**	16,70	16,73
	CE	R	**14,91**	18,10	21,19
		L	**14,80**	**14,82**	20,57
PLml (mm)	OE	R	16,25	**13,22**	17,14
		L	**14,32**	15,63	**10,84**
	CE	R	**13,10**	**12,79**	15,61
		L	**13,56**	**13,00**	**14,51**
PLcop (mm)	OE	R	17,38	**14,24**	16,24
		L	**11,47**	**14,92**	**13,10**
	CE	R	**13,14**	**14,05**	18,05
		L	**13,19**	**12,96**	16,33
MVap (mm/s)	OE	R	19,11	17,41	18,08
		L	**12,49**	16,57	16,73
	CE	R	15,01	18,15	21,18
		L	**14,79**	**14,89**	20,56
MVml (mm/s)	OE	R	16,25	**13,14**	17,09
		L	**14,38**	15,55	**10,84**
	CE	R	**13,30**	**12,83**	15,60
		L	**13,53**	**12,98**	**14,50**
MVcop (mm/s)	OE	R	17,39	**14,15**	16,18
		L	**11,50**	**14,83**	**13,09**
	CE	R	**13,23**	**14,09**	18,04
		L	**13,17**	**13,02**	16,32
SA (mm^2)	OE	R	31,34	35,25	30,73
		L	26,69	39,96	39,42
	CE	R	29,31	27,17	38,90
		L	27,42	28,50	37,08
**SLL**					
TTS (s)		R	**7,45**	**8,06**	**11,81**
		L	**5,94**	**10,06**	**10,00**
PLcop (mm)		R	**14,18**	**12,11**	**13,21**
		L	**9,25**	**13,19**	**9,05**
MVcop (mm/s)		R	**13,30**	**11,41**	**12,37**
		L	**10,08**	**12,69**	**8,95**
SA (mm^2)		R	26,72	32,30	28,09
		L	22,35	27,23	24,85

OE: Open Eyes ; CE: Closed Eyes ; L: Left leg ; R: Right leg ; SLB : Single Leg Balance ; SLL : Single Leg Landing ; PLap : anteroposterior Path Length ; PLml : mediolateral Path Length ; PLcop : Centre of Pressure Path Length ; MVap : anteroposterior Mean Velocity ; MVml : mediolateral Mean Velocity ; MVcop : centre of pressure Mean Velocity ; SA : Surface. Values highlighted in bold are those below 15%.
